# Spontaneous arising of a lymphoblastoid B‐cell line harbouring a pre‐leukemic DNMT3A mutation in acute myeloid leukaemia cell culture

**DOI:** 10.1111/jcmm.16992

**Published:** 2021-10-14

**Authors:** Michal Hayun, Moran Szwarcwort, Dina Rosenberg, Dvora Sahar, Yishai Ofran

**Affiliations:** ^1^ The Clinical Research Institute at Rambam (CRIR) Rambam Health Care Campus Haifa Israel; ^2^ Virology Laboratory Rambam Health Care Campus Haifa Israel; ^3^ Hematology Laboratory Rambam Health Care Campus Haifa Israel; ^4^ Department of Hematology and Bone Marrow Transplantation Rambam Health Care Campus Haifa Israel; ^5^ Department of Hematology Shaare Zedek Medical Center Faculty of Medicine the Hebrew University of Jerusalem Jerusalem Israel

**Keywords:** acute myeloid leukaemia, B‐cell functions, DNMT3A, pre‐leukemic mutations, spontaneously arising lymphoblastoid cell line

## CONFLICT OF INTEREST

The authors have no conflicts to declare.

## AUTHOR CONTRIBUTIONS

**Michal Hayun:** Conceptualization (lead); Formal analysis (lead); Methodology (lead); Project administration (equal); Writing‐original draft (lead); Writing‐review & editing (equal). **Moran Szwarcwort:** Data curation (supporting); Formal analysis (supporting); Investigation (supporting); Writing‐original draft (supporting); Writing‐review & editing (supporting). **Dina Rosenberg:** Data curation (supporting); Formal analysis (supporting); Methodology (supporting); Writing‐original draft (supporting); Writing‐review & editing (supporting). **Dvora Sahar:** Data curation (supporting); Formal analysis (supporting); Methodology (supporting); Writing‐original draft (supporting); Writing‐review & editing (supporting). **Yishai Ofran:** Conceptualization (supporting); Project administration (equal); Supervision (lead); Writing‐original draft (supporting); Writing‐review & editing (equal).

Pre‐leukemic mutations (e.g., DNMT3A), first discovered in lymphocytes of patients with acute myeloid leukaemia (AML), occur very early during leukemogenesis at the stem cell level.^1^ There is paucity of data about the effect of these mutations on lymphocyte functions in AML, mainly due to the lack of an appropriate model to study this phenomenon. We herein report on spontaneous generation of lymphoblastoid cell lines (SP‐LCLs) that originated from peripheral blood mononuclear cells (PBMCs) of EBV‐seropositive AML patients. One of the SP‐LCLs carried DNMT3A mutation.

Epstein‐Barr virus (EBV), a DNA herpesvirus, latently residing in lymphocytes, can immortalize normal B cells in vitro and lead to lymphoblastoid cell line (LCL) development. Such immortalization of resting B cells gives rise to an actively proliferating population, usually resulting in polyclonal activation of B‐lymphocytes.^2^ Approximately 95% of adults are exposed to EBV, mainly during childhood, and EBV‐induced B‐cell malignancies (e.g., Burkitt lymphoma, Hodgkin lymphoma) are well‐documented.^3^

To stimulate B‐cell growth from PBMCs of either EBV‐positive patients or healthy EBV‐seropositive donors to establish an LCL in vitro, the use of cyclosporine A, as a selective immuno‐suppressive agent, is required.^4^ However, spontaneous generation of LCLs (SP‐LCLs) from PBMCs, without EBV infection or exposure to transforming agents, as observed in our study, is rare. Previously reported spontaneous appearance of immortalized B‐cell lines originated from PBMCs of patients with active autoimmune diseases, including Sjogren's syndrome, systemic lupus erythematosus, rheumatoid arthritis^5^ or multiple sclerosis.^6^ To the best of our knowledge, this is the first report of spontaneous outgrowth of LCLs in culture of myeloid blasts obtained from AML patients.

**FIGURE 1:**
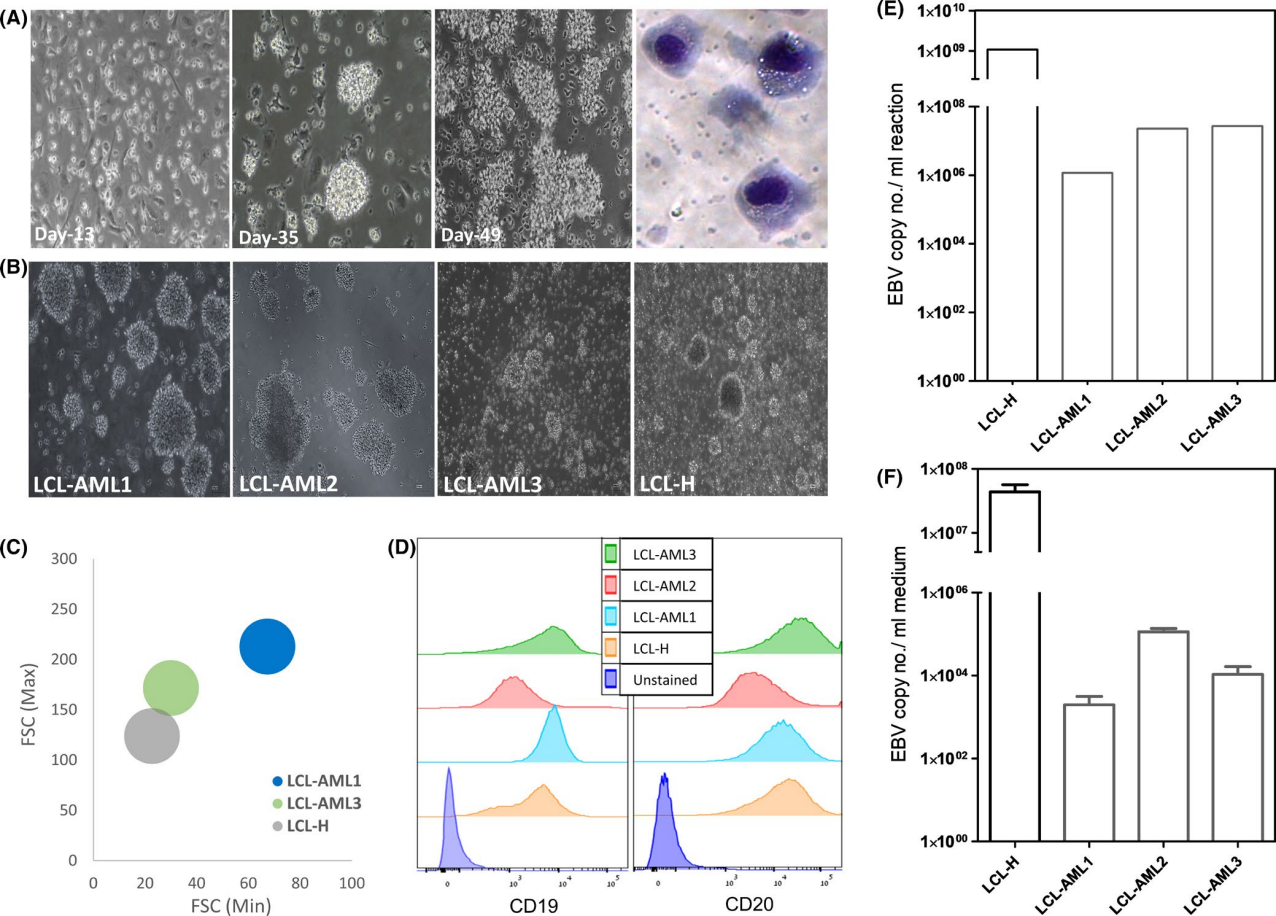
Identification of SP‐LCLs originated from PBMCs of AML patients using different methods. (A) Morphological changes occurring during SP‐LCL development in culture. The transition from a single‐cell suspension of AML blasts in culture through rosette colonies to big clumps is demonstrated. The following method was used to obtain these SP‐LCLs: PBMCs were drawn from AML patients at diagnosis and the mononuclear cells were separated by centrifugation over a layer of LymphoprepTM (Axis‐Shield PoC AS, Oslo, Norway). After washing with PBS, 1.5 × 10^6^ PBMCs/ml were seeded in a T75 flask containing RPMI1640 medium supplemented with 15% FBS, 2 mM L‐glutamine and 1% penicillin‐streptomycin and incubated at 37°C in 5%CO_2_. Cells were followed using inverted microscopy (×20 magnification) until the rosette‐like formation was observed. Cytospin preparation of SP‐LCLs obtained from an AML patient was stained using Wright‐Giemsa stain. The image shows the cells with a high cytoplasmic/nuclear ratio and cytoplasmic vacuoles (light microscopy, ×100 magnification). (B) SP‐LCLs (LCL‐AML1, LCL‐AML2 and LCL‐AML3) and an LCL established from healthy donor PBMCs (LCL‐H) appearing in the cultures (passages 5–35; inverted microscopy, ×10 magnification). (C) Schematic presentation of an LCL size measured by flow cytometry (forward scatter, FSC and side scatter, SSC). Results represent an average size of the bulk cells, calculated based on three cell‐passage measurements for each LCL. (D) Expression levels of B‐cell markers on SP‐LCLs and LCL‐H, evaluated by flow cytometry, using PE‐anti‐CD19 and APC‐anti‐CD20 antibodies. (E) EBV‐DNA detection in SP‐LCL and LCL‐H cultures: DNA was extracted from LCLs (Exgene cell SV kit, GeneAll Biotechnology, Korea) and EBV‐DNA was detected using quantitative real‐time PCR (Rotor‐Gene 6000 cycler). The total reaction volume contained nucleic acid extraction and PCR primers amplifying the membrane protein BNRF1 p143. The range of EBV copy numbers in SP‐LCL was 10^6^–10^7^, while 10^9^ copies/ml were observed in LCL‐H. (F) EBV‐DNA was detected in culture supernatants collected from SP‐LCLs and LCL‐H and quantified by real‐time PCR. The range of EBV copy numbers in SP‐LCL was 10^3^–10^5^, while 10^7.5^ copies/ml were observed in LCL‐H

To culture AML blasts, eight blood samples were drawn from AML patients at diagnosis (Table S1), processed and cultured. The viability and ability of the cell cultures to grow were routinely examined with microscopy, without splitting the cells or changing the growth media. Within two weeks, most cells died, while a minor cell population adhered to the culture flask and was recognized as stroma cells. Remarkably, in three of the eight samples, 15% of the cells remained alive and were later identified as lymphocytes. During the third week, live single suspended cells and several small cell clusters repopulated the culture, constituting 30% of the total cell population. A week later, the cells duplicated and the clusters enlarged, while exhibiting rosette‐like shape morphology. In immunophenotyping, 80% of these cells displayed the B‐lymphocyte phenotype. At weeks 5–6, Giemsa staining revealed numerous vacuoles in the cytoplasm. The process of blast culture transition to a continuously proliferating LCL is demonstrated in Figure 1A. B‐LCLs (LCL‐AML1, LCL‐AML2, LCL‐AML3), originating from three EBV‐seropositive AML patients, spontaneously grew in the culture. These SP‐LCL clusters appeared to be very dense, tightly packed and formed large clumps (Figure 1B). Surprisingly, none of the cell surface antigens defining AML blasts (CD34, CD117, or CD33) was expressed. We found associations of high blood cell counts and a high blast percentage with spontaneous generation of LCLs, without correlation to the mutation profile (Table S1). More patient‐derived SP‐LCLs are required to confirm these data.

EBV‐transformed human B‐LCLs can be established by the traditional method using EBV infection of PBMCs isolated from healthy donor blood (LCL‐H).^4^ We created such cells to serve as a reference for the characterization of the SP‐LCLs produced in our study. SP‐LCLs were found to be larger in size than LCL‐H, which was particularly evident in LCL‐AML1 (*p *< 0.01) (Figure 1C). Additionally, similar to LCL‐H, all SP‐LCLs expressed the pan B‐cell markers CD19 and CD20 (Figure 1D).

The quantitative EBV RT‐PCR assay revealed a range of 10^6^–10^7^ DNA copies in SP‐LCLs, while this number was as high as 10^9^ in LCL‐H (Figure 1E). Likewise, significantly higher levels of EBV‐DNA were released to the culture media by LCL‐H compared with SP‐LCLs (>2 logs) (Figure 1F). The low EBV copy number was needed to obtain a rapidly proliferating SP‐LCL culture compared with the designed LCL‐H, which may be explained by reactivation of latent EBV under stress conditions in the culture.

**FIGURE 2:**
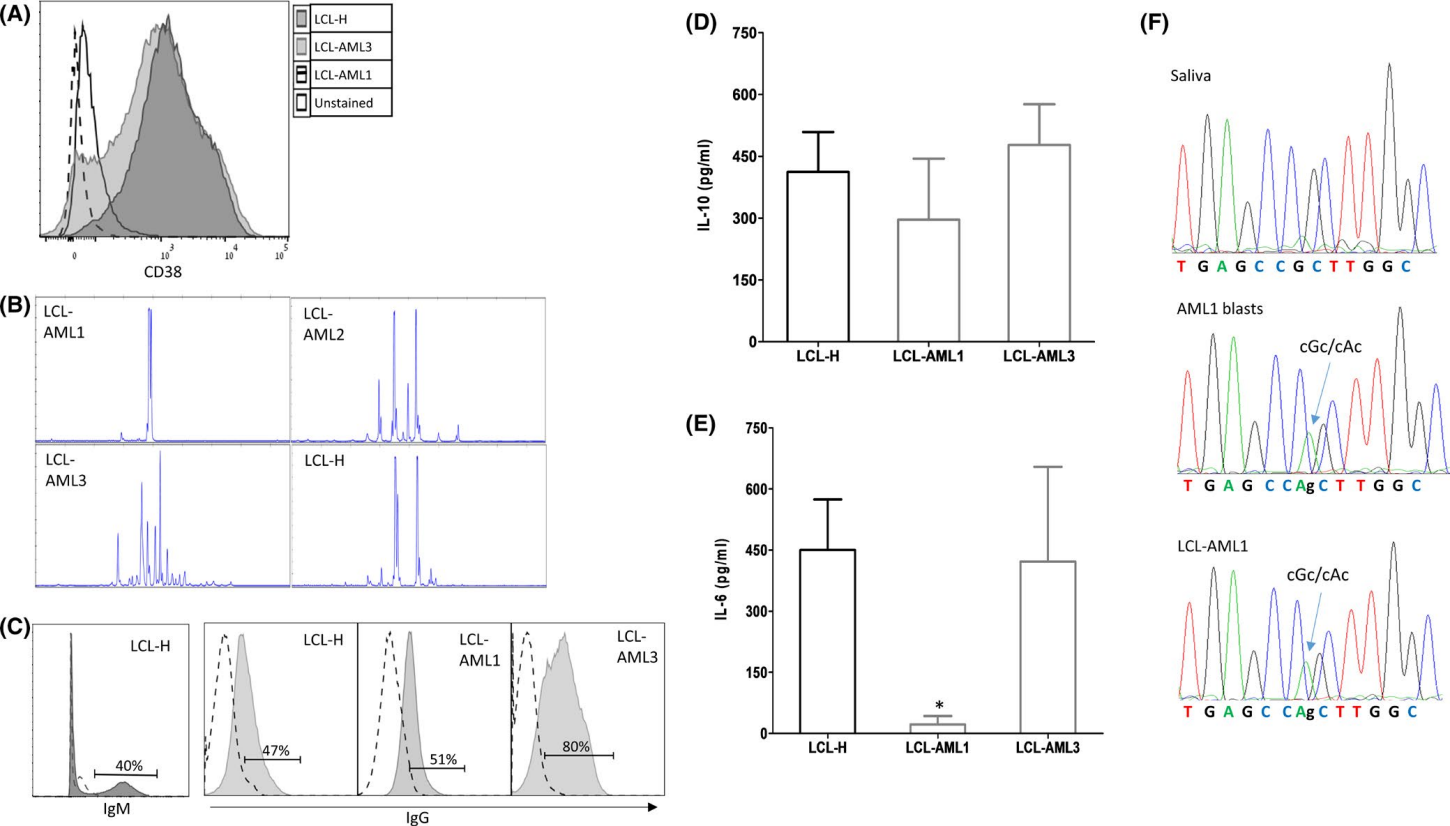
Genetic and Functional Features of SP‐LCLs. (A) Maturation stage of the B cells, originating from the LCL cultures was examined by immunophenotyping. The immunophenotype of LCL‐H and LCL‐AML2 was identical, but differed from that of LCL‐AML1 in the ability to express CD38. (B) Clonality pattern of the LCLs: IgH B‐cell clonality as assessed by IdentiClone™ IgH Gene Clonality Assay (InVivoScribe Technologies): a reaction of multiplex PCR followed by fragment size analysis of fluorescently labelled products using Genetic Analyzer. (C) Cell surface expression of the heavy chain expressed on LCLs was detected by flow cytometry using FITC‐anti‐IgG and APC‐anti‐IgM antibodies. (D–E) Cytokines secreted from SP‐LCLs (LCL‐AML1 and LCL‐AML3) and LCL‐H to the culture media. IL‐10 and IL‐6 secreted levels were measured in the supernatants by ELISA MAX™ (BioLegend, San Diego, CA). Results represent an average of 4 repeats (ANOVA test, *p < 0.05). (F) Detection of a single mutation in DNMT3A at R882H both in blast cells obtained at diagnosis and in the LCL‐AML1, but not in the saliva derived from this patient. Sanger sequencing with oligonucleotide primers for DNMT3A (forward‐ 5′‐ TTTTCTCCCCCAGGGTATTT‐3′, revers‐ 5′‐GAAGAGGTGGCGGATGACT‐3′) was performed using 3730 DNA Analyzer (Applied Biosystems, Carlsbad, CA)

The ability to produce and secrete immunoglobulins is known to depend on the maturation stage of B cells. We identified surface marker phenotypes of LCL‐H, LCL‐AML2 and LCL‐AML1 as CD19^+^CD20^+^CD10^−^CD5^−^CD38^+^CD27^+^IgD^−^, CD19^+^CD20^+^CD10^−^CD5^−^CD38^+^CD27^+/−^IgD^−^ and CD19^+^CD20^+^CD10^−^CD5^−^CD38^−^CD27^+^IgD^−^, respectively. The two former immunophenotypes represented plasmablasts (proliferating, short‐lived activated B cells), while the latter one was typical of memory B cells (long‐lived B cells). The main difference between these immunophenotypes lays in the CD38 expression (Figure 2A).

B‐cell clones are known to possess unique immunoglobulin heavy chain (IgH) gene rearrangements. LCLs were found to differ in their IgH rearrangement: monoclonality, often presented by expansion of a single B‐cell, was observed in LCL‐AML1, oligoclonality—in LCL‐AML2, polyclonality—in LCL‐AML3 and bi‐clonality was evident in LCL‐H (Figure 2B). Yet, a possibility of LCL‐AML1 malignant nature was ruled out by FISH analysis for t(8:14) (data not shown). The monoclonality pattern might be related to selection of a mutated pre‐leukemic clone. As for the cell surface protein expression, IgG immunoglobulin was detected in 51% of LCL‐AML1, 80% of LCL‐AML3 and 47% of LCL‐H, which also expressed IgM (40%) (Figure 2C).

Autocrine growth factors IL‐6 and IL‐10 are among multiple soluble cytokines, known to be produced by EBV‐immortalized B‐cell lines. These interleukins play an important role in promoting continuous proliferation of these cells in vitro.^7^, ^8^ In our study, secreted IL‐10 levels were found to be within a similar range in all the three evaluated LCLs, while IL‐6 was not detected in LCL‐AML1 (Figure 2D, 2E). The latter finding could be explained, at least in part, by the fact that LCL‐AML1 cells are highly immortalized due to their independence of the IL‐6 signalling pathway.

The DNMT3A gene, encoding DNA methyltransferase, is frequently mutated in various haematological malignancies. Between 17 and 34% of AML patients with a cytogenetically normal karyotype harbour point mutations in DNMT3A.^9^ The R882H mutation in DNMT3A is most prevalent and leads to a roughly 40%‐reduction in overall DNA methylation activity.^10^ The mechanism of EBV‐mediated transformation of PBMCs to LCLs is reported to involve promoter hypomethylation only.^11^ Remarkably, in the present study, DNMT3A gene sequencing revealed a single homozygous mutation in R882H in LCL‐AML1 (validated using molecular inversion probe technique, 100% VAF). In this patient, the mutation was detected in blasts derived at diagnosis, but not in the germline saliva sample (Figure 2F). Therefore, we assume that a somatic mutation in DNMT3A emerges early at the hematopoietic stem cell level, potentially altering the ability of lymphocytes carrying this mutation to secret IL‐6. Of note, DNMT3A overexpression is suggested to suppress IL‐6 through alterations in the status of DNA methylation in the IL‐6 promoter.^12^

To date, only two human AML cell lines with the DNMT3A R882 mutation have been described, NPM1‐mutant OCI‐AML3^13^ (R882C) and SET‐2 (R882H, heterozygous).^14^ Christopher et al have observed different methylation patterns in primary DNMT3A^mut^ AML blasts and in OCI‐AML3, implying that this cell line cannot be employed as a relevant model for exploring DNMT3A mutations.^15^ While in AML, arginine is known to be exchanged by histidine (R882H) in about 66% of R882 mutations,^10^ apart from SET‐2, no other cell lines or in vivo models applicable to the investigation of this mutation have been reported yet. The facts that LCL‐AML1 originates from B cells in the culture derived from an AML patient and that it is not considered a ‘classically transformed’ cell line make it a reliable model to explore various aspects related to DNMT3A^mut^, especially given its rare homozygous genotype. A number of studies have shown at both molecular and functional levels, a significant close correlation between LCLs and parent lymphocytes. Hence, these cell lines can serve as a source of cells in the research of novel therapeutic modalities and as a reference in genomic investigations. However, the fact that LCLs become immortalized and the biology of the cell changes should be taken into consideration when analysing the obtained findings.

In our study, LCL‐AML1, while not fully reflecting the authentic malignant somatic genome of the blasts (NPM1 driver mut is missing), does express the pre‐leukemic mutation, not existing in the germline. Recent data provide evidence that older adults who have acquired the DNMT3A mutation experience some level of immune dysfunction involving monocytes and lymphocytes that may contribute to the cardiovascular risk and immune modulation of the heart and other organs. The reported LCL‐AML1 cell line could be used in the exploration of potential mechanisms involved in these dysfunctions, could represent a useful tool for exploring B‐cell functions in the presence of DNMT3A mutation in healthy older adults, and in studies investigating patient's sensitivity to drugs targeting such mutations. We suggest using this simple, inexpensive and available technique to obtain desired SP‐LCLs, initiating with specimens containing both high WBC counts and blast percentage.

We herein present a monoclonal SP‐LCL harbouring homozygous DNMT3A^mut^, which can be employed as a model in a variety of DNA methylation studies. Its maturation stage differs from that of other SP‐LCLs or LCL‐H. This may suggest an early selection mechanism of mutated pre‐leukemic B cells. While the exact trigger activating EBV in the rare B cells existing in the evaluated blast samples is unclear, we assume that a combination of functionally inactivated T cells and stress conditions (nutrient depletion, apoptosis of blast populations and oxidation products) in the cell culture may give growth advantage to EBV‐latent B cells.

## AVAILABILITY OF BIOLOGICAL MATERIALS

We declare that we allow to Cancer Research Technology Ximbio (ximbio.com) to provide LCL‐AML1 (=MHYO‐LCL1) and LCL‐AML3 (=MHYO‐LCL2) upon request.

## Supporting information

Table S1

## Data Availability

The data that support the findings of this study are available from the corresponding author upon reasonable request.
