# Supplementary material for: Spontaneous arising of a lymphoblastoid B‐cell line harbouring a pre‐leukemic DNMT3A mutation in acute myeloid leukaemia cell culture
Source: J Cell Mol Med. 2021 Oct 14;25(22):10778–82. doi: 10.1111/jcmm.16992 (PMC8581312; doi:10.1111/jcmm.16992)
Supplement: Supplementary file 1 — Table S1 [file JCMM-25-10778-s001.doc]

| **Pt. no.** | **Gender** | **Age at diagnosis** | **Disease state** | **EBNA IgG** | **VCA IgG** | **Mutational**  **profile** | **WBC**  **count** | **Lymphocytes (%)** | **Blasts (%)** |
| --- | --- | --- | --- | --- | --- | --- | --- | --- | --- |
| **LCL AML1** | m | 38 | Diagnosis | pos | pos | NPM1^+^ | 166,000 | 4 | 80 |
| **LCL AML2** | m | 34 | Diagnosis | pos | ND | Inv.16  *Jak2^mut^ 3% | 290,000 | 6 | 65 |
| **LCL AML3** | f | 40 | Diagnosis | pos | ND | NPM1^+^  *EZH2 2% | 77,000 | 6.3 | 33 |
| **4** | f | 66 | Diagnosis | pos | ND | FLT3-ITD | 35,450 | 9.8 | 90 |
| **5** | m | 66 | Diagnosis | NA | NA | NPM1^+^ | 8,540 | 16.6 | 40 |
| **6** | f | 69 | Diagnosis | pos | ND | - | 5,920 | 51 | 52 |
| **7** | m | 42 | Diagnosis | pos | ND | NPM1^+^ FLT3-ITD | 226,000 | 5.2 | 28 |
| **8** | f | 68 | Diagnosis | NA | NA | FLT3-ITD  FLT3 TKD (D835) | 176,000 | 40.6 | 66 |

**Supplementary Table 1S. Patient Characteristics**

m: male; f: female; pos: positive; NA: not available.; ND: not detected.; WBC: white blood cell; *: evaluated using the molecular inversion probe technique
